# Antimutagenic, anti-inflammatory, and antioxidative activities of the juice of *Vitis ficifolia* var. *Ganebu*, a woody vine in the grape family, known as Ryukyu-ganebu in Japan

**DOI:** 10.1186/s41021-021-00225-y

**Published:** 2021-11-12

**Authors:** Sakae Arimoto-Kobayashi, Ryoko Hida, Nana Fujii, Ryosuke Mochioka

**Affiliations:** 1 Graduate School of Medicine, Dentistry and Pharmaceutical Sciences, Okayama University, 700-8530 Okayama, Japan; 2 Faculty of Pharmaceutical Sciences, Okayama University, 700-8530 Okayama, Japan; 3 Faculty of Agriculture, University Farm, Kagawa University, 769-2304 Kagawa, Japan

**Keywords:** Antimutagenesis, Anti-inflammation, Acute edema, *In vivo* anti-lipid peroxidation, Wild grape juice, Ryukyu-ganebu

## Abstract

**Background:**

Mutation, inflammation, and oxidative damage including lipid-peroxidation are factors involved in the development of cancer. We investigated the antimutagenic, *in vivo* and *in vitro* anti-inflammatory, and antioxidative effects of the juice of *Vitis ficifolia* var. *ganebu* (known as Ryukyu-ganebu in Japan) harvested in Kuchinoshima island (hereafter, the juice is referred to as ganebu-K) in comparison with the juice of *Vitis coignetiae* (crimson glory vine, known as yamabudo in Japan; hereafter, the juice is referred to as yamabudo) which we found antimutagenic and anti-inflammatory effects.

**Results:**

Ganebu-K inhibited the mutagenic activity of several carcinogens, MeIQx, IQ, Trp-P-2(NHOH), and MNNG, model compounds of tumor initiation. Using *S. typhimurium* YG7108, a strain lacking *O*^6^-methylguanine DNA methyltransferases, ganebu-K showed no significant inhibition of the mutagenicity of MNNG. Thus, DNA repair of O^6^-methylguanine produced by MNNG might be an antimutagenic target of the components in ganebu-K. Topical application of ganebu-K to the dorsal sides of mice resulted in potent suppression of acute edema induced by 12-*O*-tetradecanoylphorbol-13-acetate (TPA). Ganebu-K, but not yamabudo, exhibited significant inhibition of the induction of prostaglandin E_2_ (PGE2) induced by TPA. Components contained in ganebu-K, but not in yamabudo, might be responsible for the inhibition of the induction of PGE2. Ganebu-K inhibited *in vivo* lipid peroxidation and decreased the level of glutamic oxaloacetic transaminase induced by CCL_4_ treatment.

**Conclusions:**

These results suggest that the active components in ganebu-K juice are not the same as those in yamabudo, and the components in ganebu-K are attractive candidates as chemopreventive agents.

## Introduction

Dietary factors can substantially influence cancer risk in humans [1]. Plant metabolites like polyphenols and flavonoids have antioxidant and antiproliferative properties, and can be found in foods such as berries, citrus fruits, tea, and red wine. Fruits and vegetables that contain carotenoids and other antioxidants have been hypothesized to decrease lung cancer risks [2, 3]. Chemopreventive effects of cyanidin-3-glucoside from blackberry [4], proanthocyanidin extract from grape seed [5], and plant polyphenols [6] on various cancers have been reported. Previously, we found antimutagenic and anti-inflammatory effects of the juice of *Vitis coignetiae* (crimson glory vine, a deciduous vine that produces purple berries, known as yamabudo in Japan) [7, 8]. We also revealed that the oral intake of a partially purified fraction from yamabudo affords significant protection against skin and lung carcinogenesis in mice. We isolated and identified the non-polyphenolic compound 2,6-dimethoxy-1,4-benzoquinone (DBQ) from yamabudo as an anti-inflammatory substance, and found that it affords significant protection against a tobacco-specific nitrosamine, 4-(methylnitrosamino)-1-(3-pyridyl)-1-butanone (NNK), in a mouse model of lung tumorigenesis [9, 10]. Yamabudo is a wild grape, and seven species and eight varieties of wild grapes including yamabudo are known in Japan [11], but the biological activities of wild grapes other than yamabudo have not been investigated. *Vitis ficifolia* var. *ganebu* (known as Ryukyu-ganebu in Japan, and hereafter referred to as Ryukyu-ganebu) is a wild grape that originates in Japan. It is an evergreen vine that produces purple berries that can be processed for juice (hereafter referred to as ganebu). Yamabudo is distributed throughout the mainland (Honshu), Hokkaido, and Shikoku in Japan; Sakhalin island in Russia; and Ulleungdo island in Korea; but not in southern western island in Japan, whereas Ryukyu-ganebu is distributed throughout the southwestern islands of Japan, i.e., Ryukyu and Yaeyama islands in Okinawa Prefecture, and Amami and Tokara islands in Kagoshima Prefecture; but not in mainland, Hokkaido and Shikoku.

Mutation, inflammation, and oxidative damage including lipid-peroxidation are factors involved in the development of cancer [1]. In the present study, we investigated the antimutagenic, anti-inflammatory, and antioxidative effects of ganebu harvested from Kuchinoshima island in comparison with yamabudo.

## Materials and methods

### Materials

*V. ficifolia* var. *ganebu* (Ryukyu-ganebu in Japanese) grows naturally in Kuchinoshima island in Kagoshima Prefecture, Japan. Kuchinoshima island of the Tokara islands is the northern limit of the natural habitat of ganebu. Fruits of ganebu grown in Kuchinoshima were harvested in 2011 and processed with a juicer. Juice (660 mL) was obtained from 1 kg of fruits. The juice was centrifuged with 3500 rpm (2000 x g) for 15 min at 20 °C, and supernatant was obtained (hereafter, the obtained supernatant is referred to as ganebu-K). As the dry weight of 1 mL of ganebu-K was 0.461 g, the concentration of ganebu-K was 461 mg/mL. Juice of the fruits of *V. coignetiae* cultivated in Hiruzen (Okayama, Japan) and harvested in 2013 was purchased in local stores in Okayama. The juice was centrifuged with 3500 rpm (2000 x g) for 15 min at 20 °C, and supernatant was obtained (hereafter, the obtained supernatant is referred to as yamabudo). As the dry weight of 1 mL of yamabudo was 0.188 g, the concentration of yamabudo was 188 mg/mL. Samples were sterilized by filtration and kept at -20 °C until use.

*Salmonella enterica* subspecies I, serovar Typhimurium (*Salmonella typhimurium*) strain TA98 [*hisD3052 ΔuvrB gal bio chl1005 rfa1001/pKM101*], TA100 [*hisG46 ΔuvrB gal bio chl1005 rfa1001/pKM101*], and TA1535 [*hisG46 ΔuvrB gal bio chl1005 rfa1001*] were gifts from Dr. Bruce N. Ames of the University of California, Berkeley [12]. *S. typhimurium* YG7108 [*hisG46 ΔuvrB gal bio chl1005 rfa1001 Δada*_*st*_::*Km*^*r*^*Δogt*_*st*_::*Cm*^*r*^], a strain lacking *O*^6^-methylguanine DNA methyltransferases, was a gift from Dr. Masami Yamada of the National Institute of Health Sciences [13]. 2-Amino-3,8-dimethyl-3* H*-imidazo[4,5-*f*]quinoxaline (MeIQx, CAS 77500-04-0), 2-amino-3-methylimidazo(4,5-*f*)quinoline (IQ, CAS 76180-96-6), 3-amino-1-methyl-5* H*-pyrido[4,3-*b*]indole (Trp-P-2, CAS 72254-58-1), carbon tetrachloride and 7,12-dimethylbenz(a)anthracene (DMBA, CAS 57-97-6), 2,6-dimethoxy-1,4-benzoquinone (DBQ) (CAS 530-55-2), and 12-*O*-tetradecanoylphorbol-13-acetate (TPA) were purchased from FUJIFILM Wako Pure Chemical Corporation (Osaka, Japan). 1-Methyl-3-nitro-1-nitrosoguanidine (MNNG, CAS 70-25-7) was purchased from Nacalai Tesque (Kyoto, Japan). *tert*-Butyl hydroperoxide (t-BHP) was purchased from Sigma-Aldrich Japan (Tokyo). A metabolically activated form of Trp-P-2, 3-hydroxyamino-1-methyl-5* H*-pyrido[4,3-*b*]indole (Trp-P-2(NHOH)), was synthesized from Trp-P-2 according to steps outlined in the literature [14]. The supernatant fraction of rat liver homogenate (S9) was prepared from male Sprague-Dawley rats that had been administered polychlorinated biphenyl (PCB54, with a chlorine content of 54 %, Tokyo Kasei, Tokyo). The protein content of the S9 fraction was 43.0 mg/mL. Other reagents were purchased from commercial sources. All experiments were carried out in accordance with the Safety Guidelines of Okayama University, and Industrial Safety and Health Act No. 71 of 2018.

### Animals

Mice (ICR Slc male) and Sprague-Dawley rats were purchased from Japan SLC, Inc. (Hamamatsu, Japan). SENCAR mice were born and reared in our laboratory. Animals were housed five per cage in the animal room and randomly separated to form treatment groups at least one week prior to commencement of the experiment. Mice had free access to pellets of murine chow (MF powder, Oriental Yeast Co. Ltd., Tokyo, Japan) and water, and were kept on a 12-h light/12-h dark cycle with optimum air changes and a constant room temperature of 20 °C. All experiments were performed in accordance with the Guidelines for Animal Experiments at Okayama University Advanced Science Research Center (Permission No. OKU-2,012,213, 2,015,045, 2,018,028, 2,018,787, and 2,021,460) based on the Act on Welfare and Management of Animals (Act of Japan, No. 105 of October 1, 1973 and Amendment of Act No. 68 of 2005) and standards relating to the Care and Keeping and Reducing Pain of Laboratory Animals (Notice of the Ministry of the Environment No. 88 of 2006).

### Antimutagenicity test

**Fig. 1:**
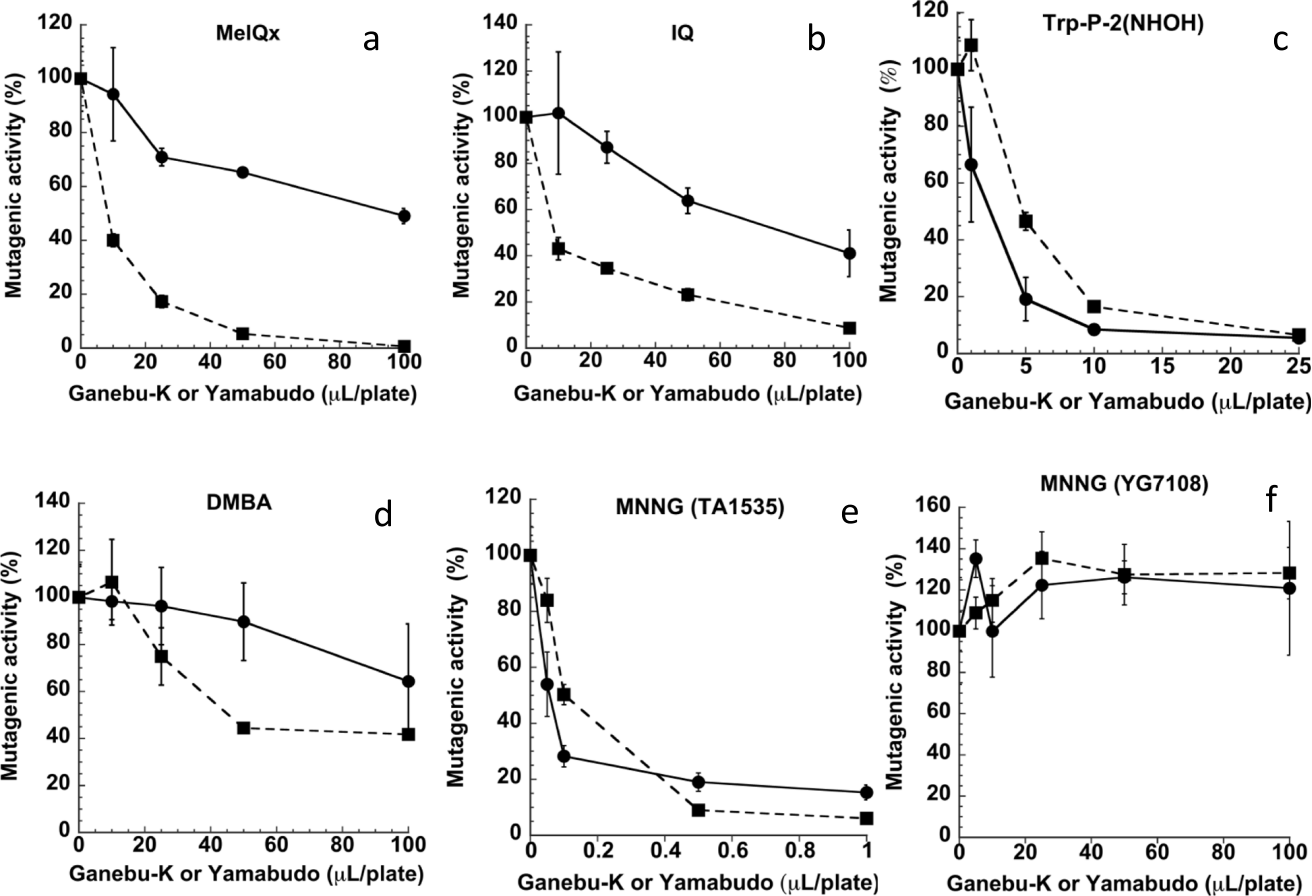
Antimutagenic activity of ganebu-K (line) and yamabudo (dotted line) towards MeIQx (**a**), IQ (**b**), Trp-P-2(NHOH) (**c**), DMBA (**d**), and MNNG (**e**-**f**) mutagenicity. MeIQx and IQ were assayed with *S. typhimurium* TA98 with +S9 (**a**-**b**), Trp-P-2(NHOH) was assayed with *S. typhimurium* TA98 with -S9 (**c**), DMBA was assayed with *S. typhimurium* TA100 with +S9 (**d**), and MNNG was assayed with *S. typhimurium* TA1535 with -S9 (**e**) and YG7108 with -S9 (**f**). SD is indicated by bars (*n *= 3)

The inhibitory effects of juice on the mutagenicity induced by MeIQx, IQ, Trp-P-2(NHOH), DMBA, and MNNG were investigated using the Ames test [12] as previously described [15]. MeIQx and IQ were assayed with *S. typhimurium* TA98 in the presence of metabolic activation with rat liver homogenate S9 (hereafter referred to as +S9). Trp-P-2(NHOH) was assayed with *S. typhimurium* TA98 in the absence of metabolic activation (hereafter referred to as -S9). DMBA was assayed with *S. typhimurium* TA100 with +S9. MNNG was assayed with *S. typhimurium* TA1535 and YG7108 with -S9. Experiments were performed in triplicate. The mutagenic activities (%) as shown in Fig. 1 were calculated as follows:

$$100\;\times\;\left[\left(\mathrm{revertants}\;\mathrm{in}\;\mathrm{the}\;\mathrm{presence}\;\mathrm{of}\;\mathrm{juice}\right)\;--\;\left(\mathrm{spontaneous}\;\mathrm{revertants}\right)\right]\;/\;\left[\left(\mathrm{revertants}\;\mathrm{in}\;\mathrm{the}\;\mathrm{absence}\;\mathrm{of}\;\mathrm{juice}\right)\;--\;\left(\mathrm{spontaneous}\;\mathrm{revertants}\right)\right].$$

### Effects on TPA-induced acute edema on the dorsal sides of mice

Juice (ganebu-K or yamabudo) was mixed with an equal volume of acetone for better permeability into skin. Six-week-old male SENCAR mice were divided into five groups each comprising five animals. The dorsal side of the skin of mice was shaved using an electric hair cutter at least one day before treatments. Samples were topically applied on a circle (2 cm in diameter) on the dorsal sides of mice. Mice in groups 1-2 received 20 µL of acetone:water (1:1), mice in group 3 received 20 µL of yamabudo, mice in group 4 received 20 µL of DBQ (1 mg/mL) dissolved in acetone:water (1:1), and mice in group 5 received 20 µL of ganebu-K. After 30 min, mice in group 1 received acetone without TPA applied on the circle, while mice in groups 2-5 each received 1.7 nmole of TPA dissolved in 20 µL of acetone applied on the circle. Thirty minutes following acetone/TPA treatment, the thickness of dorsal skin was measured with a Digital Caliper (AS ONE Corporation, Osaka, Japan). Edema was quantified as the difference of the thickness between treatments. Inhibition rate (%) of inflammation was calculated as follows:

$$100\times\left\{1-\left[\left(\mathrm{average}\;\mathrm{skin}\;\mathrm{thickness}\;\mathrm{of}\;\mathrm{groups}\;3,\;4,\;\mathrm{or}\;5\right)-\left(\mathrm{average}\;\mathrm{skin}\;\mathrm{thickness}\;\mathrm{of}\;\mathrm{group}\;1\right)\right]\;/\;\left[\left(\mathrm{average}\;\mathrm{skin}\;\mathrm{thickness}\;\mathrm{of}\;\mathrm{group}\;2\right)-\left(\mathrm{average}\;\mathrm{skin}\;\mathrm{thickness}\;\mathrm{of}\;\mathrm{group}\;1\right)\right]\right\}$$

**Fig. 2:**
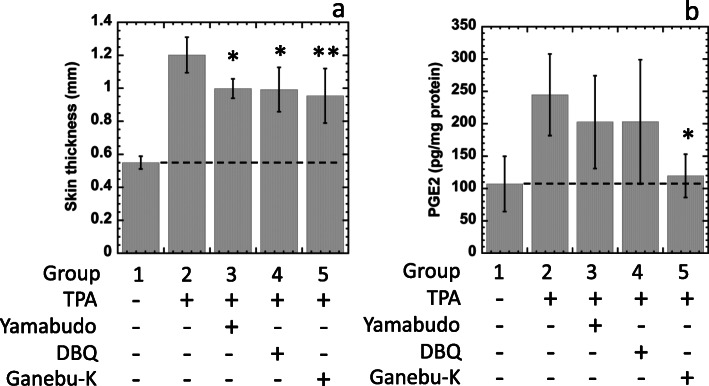
Inhibitory effects of yamabudo (group 3), DBQ (group 4), and ganebu-K (group 5) on TPA-induced inflammation of mouse skin thickness (**a**) and PGE2 induction (**b**). Results in group 1 serve as a negative control and those of group 2 as a positive control. SD is indicated by bars (*n *= 5). *p<0.05 and ** *p*<0.01, significantly different from group 2

The results of mice in group 1 represented a negative control (NC), those of group 2 indicated the effects with TPA as a positive control (PC), and those of groups 3-5 represented the effects of TPA in the presence of yamabudo (group 3), DBQ (group 4), or ganebu-K (group 5) as shown in Fig. 2a.

Prostaglandin E2 (PGE2) induction in treated skin was measured as follows. Briefly, mice were sacrificed, and circular Sect. (6 mm in diameter) in the treated circles (2 cm in diameter as described above) of treated dorsal skin were punched out using a cork borer and frozen with liquid N_2_ until use. Obtained skin was homogenized in cold 0.1 M sodium phosphate buffer (pH 7.4) containing 1 mM EDTA and 10 µg/mL indomethacin. Homogenates were centrifuged at 10,000 x g for 30 min at 4 °C, and the supernatant fraction was used for the analysis of PGE2 induction. PGE2 was measured using a commercial assay kit (Prostaglandin E2 Parameter Assay Kit, R&D Systems, MN, USA) according to the manufacturer’s instructions. Results are shown in Fig. 2b. The inhibition rates of PGE2 levels (%) were calculated similar to those formerly described.

### *In vitro* preventive effects of oxidative damage, including lipid peroxidation

**Fig. 3:**
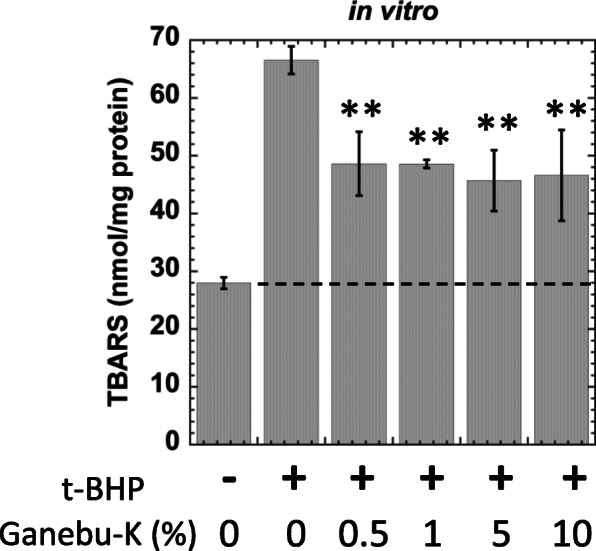
Effects of ganebu-K on *in vitro* lipid peroxidation. SD is indicated by bars (*n *= 5). ** Significantly different from second left data at *p*<0.01

Thiobarbituric acid reactive substance (TBARS) assays were used for the *in vitro* anti-lipid peroxidation study of ganebu-K. Ganebu-K was freeze-dried and dissolved and diluted in water to obtain various concentrations, 0.5 %, 1 %, 5 %, and 10 % eq. of the original juice. Livers from non-treated mice (SENCAR, male, seven weeks old, *n *= 3) were homogenized with phosphate buffer (pH 7.0, 50 mM) and centrifuged (15 min, 9000 *g*, 4 °C). The obtained liver supernatant (50 µL), t-BHP (1 M, 2 µL), and 48 µL of various concentrations of ganebu-K (0, 0.5, 1, 5, or 10 % in water, as indicated in Fig. 3) were mixed and incubated at 37 °C for 2 h. For the NC, the liver supernatant (50 µL) and solvent (50 µL) were mixed without t-BHP and incubated as above. The amounts of malondialdehyde produced were determined by the TBARS method using a TBARS assay kit (Cayman, Ann Arbor, MI, USA).

### Protective effects on carbon tetrachloride-induced hepatic dysfunction

**Fig. 4:**
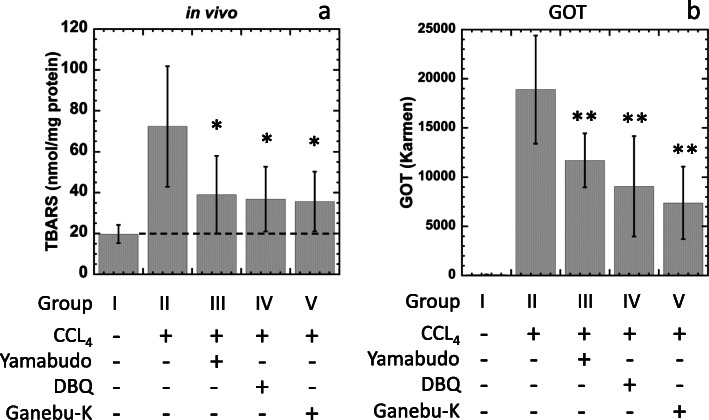
Effects of yamabudo (group 3), DBQ (group 4), and ganebu-K (group 5) on *in vivo* lipid peroxidation (**a**) and on plasma GOT levels (**b**). Results in group 1 serve as a negative control and those of group 2 as a positive control. SD is indicated by bars (*n *= 5). *p<0.05 and ** *p*<0.01, significantly different from group II

We evaluated the hepatoprotective effects against carbon tetrachloride-induced *in vivo* hepatotoxicity. Briefly, mice (ICR, six weeks old, male) were divided into five groups of five animals (groups I-V). Mice in groups I and II received 10 mL/kg of water, mice in group III received 10 mL/kg of yamabudo, mice in group IV received 10 mL/kg of DBQ (1 mg/mL dissolved in water), and mice in group V received 10 mL/kg of ganebu-K via gavage once a day for three days. Thirty minutes after the third gavage, mice were injected with 1 mL/kg of corn oil (for group I) or 1 mL/kg of 50 % carbon tetrachloride dissolved in corn oil (for groups II-V) with a single *ip* injection. Twenty-four hours after injection, mice were sacrificed, venous blood was obtained via the abdominal aorta, and livers were collected. TBARS assays were used to measure *in vivo* anti-lipid peroxidation in carbon tetrachloride-treated livers. Briefly, obtained livers were homogenized with sodium phosphate buffer (pH 7.0, 50 mM) and centrifuged (15 min, 10,000 x g, 4 °C). Supernatants were collected, and the levels of 2-thiobarbituric acid reactive substances (TBARS) were determined with a TBARS assay kit (Cayman, MI, USA) (Fig. 4a). Blood was centrifuged with heparin, and plasma was taken to determine glutamic oxaloacetic transaminase (GOT) levels (Fig. 4b). GOT was measured using an assay kit (Transaminase CII test) purchased from FUJIFILM Wako Pure Chemical Corporation. The inhibition rate of TBARS and GOT levels (%) were calculated similar to those formerly described.

### Statistical analyses

Data are expressed as means ± standard deviation for each data point as indicated in each figure. *P* values < 0.05 were considered to be statistically significant. Statistical analyses were performed using KaleidaGraph (Synergy Software, Reading, PA) and Excel add-in software, Excel statistics (SSRI Co. Ltd., Tokyo, Japan).

## Results

### Antimutagenesis study with ganebu-K and yamabudo

The mutagenicity of MeIQx and IQ detected using TA98 in the presence of S9 was inhibited in the presence of ganebu-K in a dose-dependent manner, and yamabudo also inhibited the mutagenicity of MeIQx and IQ (Fig. 1ab). The amounts (volume) of ganebu-K needed for 50 % inhibition (hereafter referred as to ID_50(V)_) of the mutagenicity of MeIQx and IQ were approximately 100 µL/plate (Fig. 1a) and 80 µL/plate (Fig. 1b), respectively. As the concentration of ganebu-K was 461 mg/mL, the amounts (weight) of ganebu-K needed for 50 % inhibition (hereafter referred as to ID_50(W)_) of the mutagenicity of MeIQx and IQ were approximately 46.1 mg/plate and 36.9 mg/plate, respectively. The ID_50(V)_ and ID_50(W)_ values for yamabudo were each approximately 10 µL/plate and 1.88 mg/plate for the mutagenicity of MeIQx and IQ (Fig. 1ab), as the concentration of yamabudo was 188 mg/mL. The mutagenicity of Trp-P-2(NHOH) detected with TA98 (-S9) was also inhibited in the presence of ganebu-K or yamabudo (Fig. 1c). The ID_50(V)_ and ID_50(W)_ for ganebu-K was approximately 2 µL/plate and 0.922 mg/plate, and those for yamabudo was approximately 5 µL/plate and 0.94 mg/plate (Fig. 1c). The mutagenicity of DMBA detected using TA100 with +S9 was inhibited in the presence of yamabudo, but not significantly inhibited in the presence of ganebu-K (Fig. 1d). The ID_50_ for yamabudo was approximately 40 µL/plate and 7.52 mg/plate against the mutagenicity of DMBA (Fig. 1d). The mutagenicity of MNNG detected with TA1535 (-S9) was inhibited in the presence of ganebu-K and yamabudo in a dose-dependent manner, respectively (Fig. 1e). The ID_50_ against the mutagenicity of MNNG detected with TA1535 (-S9) was approximately 0.050 µL/plate and 23.1 µg/plate for ganebu-K, and 0.1 µL/plate and 18.8 µg/plate for yamabudo. However, the mutagenicity of MNNG detected using YG7108 with -S9 was not inhibited in the presence of ganebu-K or yamabudo up to 100 µL/plate (Fig. 1f).

The number of His^+^ revertants per plate in the absence of ganebu nor yamabudo (100 % values of mutagenicity) was 1242 ± 82 for 200 pmole of MeIQx (TA98, +S9), 1394 ± 442 for 80 pmole of IQ (TA98, +S9), 2700 ± 301 for 20 pmole of Trp-P-2(NHOH) (TA98, -S9), 1697 ± 213 for 50 nmole of DMBA (TA100, +S9), 3713 ± 321 for 4 nmole of MNNG (TA1535, -S9), and 1043 ± 193 for 0.1 nmole of MNNG (YG7108, -S9). The number of spontaneous His^+^ revertants per plate (negative control) found in the absence of ganebu, yamabudo, and mutagens was 21.3 ± 12.1 for TA98 (+S9), 33.9 ± 11.3 for TA98 (-S9), 135.3 ± 41.2 for TA100 (+S9), 8.6 ± 3.4 for TA1535 (-S9), and 12.0 ± 3.6 for YG7108 (-S9).

### Effects on TPA-induced acute edema on dorsal side of mouse

Since ganebu-K inhibited the mutagenicity of the carcinogens above mentioned (Fig. 1), we further investigated its biological activities. The anti-inflammatory activity of ganebu-K as well as yamabudo and DBQ were evaluated in a mouse model (Fig. 2). The skin thicknesses of mice treated with TPA (group 2) were significantly increased compared with mock-treated skin of mice in group 1 (Fig. 2a), so a single topical application of TPA induced dorsal edema in mice. TPA-induced edema of mice on the dorsal side was significantly inhibited with topical application of yamabudo (group 3), DBQ (group 4), and ganebu-K (group 5) from group 2, respectively. Average rates of the inhibition of the inflammation were 31.3 ± 9.0 % (group 3), 32.2 ± 20.6 % (group 4), and 38.0 ± 25.4 % (group 5), respectively (Fig. 2a). Statistical analysis was performed with Dunnett test following one-way ANOVA (Fig. 2ab). Two-sided test was performed.

The amounts of PGE2 in TPA-treated skin of mice in group 2 were significantly increased compared with mock-treated skin of mice in group 1 (Fig. 2b). Topically applied ganebu-K, but not yamabudo or DBQ, significantly inhibited the PGE2 induction of TPA treatment compared with group 2. The inhibition rate of PGE2 induction (%) in the presence of ganebu-K was calculated as 91.0 % ± 24.3 % (Fig. 2b).

### *In vitro* lipid peroxidation study

We investigated the preventive effects of ganebu-K on oxidative damage, including lipid peroxidation. TBARS levels in liver supernatants were increased by treatment with *t*-BHP. In the presence of ganebu-K (from 0.5 to 10 % eq. of the original juice), TBARS levels in the supernatants of *t*-BHP-treated livers significantly decreased by 50 % (Fig. 3). No dose-responsible effects of ganebu-K on TBARS levels was observed. Statistical analysis was performed with Dunnett test following one-way ANOVA and significant difference from positive control (+ *t*-BHP & 0 % ganebu-K) was analyzed.

### *In vivo* protective effects against carbon tetrachloride-induced hepatic dysfunction

We investigated the preventive effects on *in vivo* oxidative damage, including lipid peroxidation, caused by CCL_4_ toxicity. TBARS levels in the liver were increased with CCL_4_ injection (group II) compared with the mock-injected group (group I) (Fig. 4a). TBARS levels in the livers of mice injected with CCL_4_ and orally administered yamabudo (group III), DBQ (group IV), or ganebu-K (group V) were significantly decreased compared with group II (Fig. 4a). The inhibition rates (%) of TBARS levels after oral administration of yamabudo, DBQ, or ganebu-K were calculated as 63.3 %, 67.5 %, and 69.8 %, respectively. GOT levels in blood plasma were also increased with CCL_4_ injection (group II) compared with the mock-injected group (group I) (Fig. 4b). GOT levels of mice injected with CCL_4_ and orally given yamabudo (group III), DBQ (group IV), or ganebu-K (group V) were significantly decreased compared with group II (Fig. 4b). The inhibition rates (%) of GOT levels with oral administration of yamabudo, DBQ, or ganebu-K were calculated as 38.2 %, 40.5 %, and 61.2 %, respectively. Statistical analysis was performed with Dunnett test following one-way ANOVA and significant difference from group 2 was analyzed.

## Discussion

We investigated the biological activities of ganebu-K in comparison with yamabudo. As a working hypothesis, we supposed that ganebu-K has antimutagenic, anti-inflammatory, and antioxidative activities similar to yamabudo. We investigated the antimutagenic effects of ganebu juice on well-known mutagenic carcinogens such as heterocyclic amines (MeIQx, and IQ), an activated heterocyclic amine (Trp-P-2(NHOH)), a polyaromatic hydrocarbon (DMBA), and an alkylating agent (MNNG) using the Ames test. The results showed that ganebu-K juice inhibited the mutagenicity of MeIQx and IQ, but not DMBQ, in the presence of metabolic activation. MeIQx, IQ, and DMBQ require metabolic activation to be mutagenic [16, 17]. Previously, we found that yamabudo decreased the activities of phase I enzymes (EROD and MROD) and increased the activities of phase II bio-inactivation enzymes (UGT and GST) [7]. MeIQx and IQ undergo N-hydroxylation by P450 1A2 as a first step of metabolic activation, and DMBA is metabolized to oxidized forms, for example epoxide and diol derivatives, by P450 1B1 [18]. Modulation of metabolic enzymes could be one antimutagenic mechanism against the genotoxicity of MeIQx, IQ, and DMBQ. The ID_50(W)_ values of ganebu-K against the mutagenicity of MeIQx and IQ were approximately twenty-five and twenty times higher than those of yamabudo, indicating that the concentration of antimutagenic components in ganebu-K might be one-25th or one-twentieth that of yamabudo (Fig. 1ab). The concentration of antimutagenic components in ganebu-K might be insufficient for antimutagenicity towards DMBA (Fig. 1d). Ganebu-K juice also inhibited the mutagenicity of an *N*-hydroxylated heterocyclic amine, Trp-P-2(NHOH), in the absence of metabolic activation (Fig. 1c). Degradation of Trp-P-2(NHOH) is stimulated in the presence of purpurin [19] and superoxide dismutase [20]. The components in ganebu-K, as well as yamabudo, might accelerate the degradation of Trp-P-2(NHOH) to reduce the mutagenicity of Trp-P-2(NHOH). The ID_50_ of ganebu-K for Trp-P-2(NHOH), 0.922 mg/plate, was much smaller than those for MeIQx and IQ, at 46.1 mg/plate. Lower amounts of components in ganebu-K might be sufficient for the degradation of Trp-P-2(NHOH) compared with the suppression of S9 enzymes required for the mutagenicity of MeIQx and IQ.

We investigated whether ganebu can inhibit the mutagenicity of an alkylating agent, MNNG. The mutagenicity of MNNG detected using TA1535 was decreased in the presence of ganebu-K (Fig. 1e). The ID_50_ value for ganebu-K, 0.050 mL and 23.1 mg, was similar to that for yamabudo, at 0.1 mL and 18.8 mg. As MNNG is a direct-acting mutagen, mechanisms for antimutagenicity have no relation to its metabolic activation. MNNG begins its actions by forming methyl-adducts in DNA. If these are not removed, the adducts mispair with the wrong base during DNA replication, resulting in mutation. We examined whether the repair systems for methyl-adducts in DNA were targets of the antimutagenic components in ganebu-K. YG7108 is a derivative of the Ames tester strain TA1535, but has chromosomal deletions of both the ogt^ST^ and ada^ST^ genes encoding *O*^6^-methylguanine DNA methyltransferases [13]. MNNG mutagenicity detected with *S. typhimurium* YG7108 was not decreased or increased in the presence of ganebu-K up to 100 µL/plate (Fig. 1f). Thus, *O*^6^-methylguanine DNA methyltransferases might be a target of the antimutagenic components in ganebu-K, which might accelerate the repair process, specifically enhancing the repair of DNA damage caused by MNNG and reducing cellular DNA damage [10].

We investigated the anti-inflammatory activity of ganebu-K. Topical application of ganebu-K to mouse dorsal sides resulted in potent suppression of acute edema induced by TPA (Fig. 2a), as did yamabudo and DBQ. To further investigate the mechanism of the anti-inflammatory activity of ganebu-K, the effect on PGE2 induction in mouse skin treated with TPA was investigated. In experiments involving topical application, as shown in Fig. 2b, ganebu-K significantly inhibited the induction of PGE2 induced by TPA. No significant inhibition of PGE2 induction was observed with yamabudo. Some particular components in ganebu-K that are not present in yamabudo might inhibit the induction of PGE2. These results also suggest that components in ganebu-K can be transcutaneously imported to target cells and eventually lead to the suppression of the inflammatory response via suppression of PGE2 induction.

Fruits and vegetables that contain antioxidants have been hypothesized to decrease the risk of various diseases, including cancer. The relationship between the antioxidant and anticancer activities of plant polyphenols, flavonoids, and anthocyanins in berries and grapes is well known [21, 22]. A plant polyphenol, resveratrol, has been proven to have antioxidant, anti-inflammatory, and anticancer effects [23], and the antioxidant properties of components in yamabudo seeds have been reported [24]. We investigated the antioxidant activities of ganebu-K juice using an *in vitro* lipid peroxidation assay. We found that ganebu-K significantly decreased lipid peroxidation (Fig. 3). In our previous study, *in vitro* lipid peroxidation was inhibited in the presence of 5-10 % eq. of the original yamabudo juice [10], but a smaller amount of ganebu-K (0.5 %) showed effective antioxidative activity. In our previous research [10], TBARS level in the supernatant of t-BHP-treated liver decreased dose-dependently to the level of the negative control in the presence of yamabudo (10 %). However, in the presence of ganebu-K (from 0.5 to 10 %), TBARS levels in the supernatants of t-BHP-treated livers significantly decreased by 50 % but not to the level of negative control and no dose response was observed. There might be another components in ganebu-K conflict with antioxidative components. Petroski and Minich [25] discussed whether plant-food consumption are beneficial because of the various ‘anti-nutrient’ compounds they contain. Lectins were suggested to cause inflammation [25]. Similar ‘anti-nutrient’ components in Ganebu-K might be implicated in TBARS level. Both ganebu-K and yamabudo inhibited *in vivo* lipid peroxidation and decreased the GOT level induced by CCL_4_ treatment (Fig. 4). A non-polyphenolic component in yamabudo, DBQ, inhibited *in vivo* lipid peroxidation and decreased the GOT level induced by CCL_4_ treatment. Lipid peroxidation is an indicator of oxidative stress caused by reactive oxygen species (ROS), which play a critical role in cell death and oxidative cell damage [26]. Previously, we found that oral intake of yamabudo or DBQ affords significant protection against lung and skin carcinogenesis in mice, and antioxidant and anti-inflammatory activities were observed as possible antitumorigenic mechanisms [10]. As shown in Figs. 2, 3 and 4, ganebu-K also had *in vivo* antioxidant and anti-inflammatory activities.

In conclusion, the present study demonstrated that ganebu-K inhibited the mutagenic activity of several carcinogens that are model compounds of tumor initiation. Ganebu-K also inhibited the induction of acute inflammation in a mouse model and reduced lipid peroxidation *in vitro* and *in vivo*. Ganebu-K may have unique components that are not in yamabudo that are responsible for the inhibition of PGE2 induction. Components in ganebu-K juice are not the same as those in yamabudo. Unfortunately, antimutagenic and anti-inflammatory substances in ganebu-K are not identified. Investigation of the types and structures of antimutagenic and anti-inflammatory constituents in ganebu-K and compare the amounts of them is worth for further investigations. Our results suggest that components in ganebu-K juice are attractive candidates for chemopreventive agents.

## Data Availability

Not applicable.

## Abbreviations

MeIQx: 2-Amino-3,8-dimethyl-3*H*-imidazo[4,5-*f*]quinoxaline
IQ: 2-Amino-3-methylimidazo(4,5-*f*)quinoline
Trp-P-2: 3-Amino-1-methyl-5*H*-pyrido[4,3-*b*]indole
t-BHP: tert-Butyl hydroperoxide
DBQ: 2,6-Dimethoxy-1,4-benzoquinone
DPPH: 1,1-Diphenyl-2-picrylhydrazyl
DMBA: 7,12-Dimethylbenzoanthracene
GOT: Glutamic oxaloacetic transaminase
Trp-P-2(NHOH: 3-Hydroxyamino-1-methyl-5*H*-pyrido[4,3-*b*]indole
MNNG: 1-Methyl-3-nitro-1-nitrosoguanidine
PGE2: Prostaglandin E2
S9: Supernatant fraction of rat liver homogenate
TPA: 12-*O*-tetradecanoylphorbol-13-acetate
TBARS: Thiobarbituric acid reactive substance
